# A Potential Window onto Early Pancreatic Cancer Development: Evidence of Cancer Stem Cell Growth after Exposure to Cadmium Chloride *in Vitro*

**DOI:** 10.1289/ehp.120-a363a

**Published:** 2012-08-31

**Authors:** Julia R. Barrett

**Affiliations:** Julia R. Barrett, MS, ELS, a Madison, WI–based science writer and editor, has written for *EHP* since 1996. She is a member of the National Association of Science Writers and the Board of Editors in the Life Sciences.

Cancer stem cells are a small subset of tumor cells that are postulated to underlie tumor initiation, growth, and metastasis. Research suggests that these cells may have begun as normal stem cells that underwent mutation or other changes that derail cellular programming and alter growth control pathways. Investigating a previously established link between cadmium exposure and pancreatic cancer, a new study finds that normal human pancreatic cells chronically exposed *in vitro* to a low level of cadmium chloride acquired cancer cell characteristics and generated what appeared to be cancer stem cells **[*EHP* 120(9):1265–1271; Qu et al.]**.

Normal human pancreatic ductal epithelial cells were cultured with or without 1.0 µM of cadmium chloride for 29 weeks. The treated cells were sampled periodically and assessed for cancer cell characteristics, including increased secretion of matrix metalloproteinase-9 (MMP-9), increased invasiveness, increased anchorage-independent growth (i.e., ability to grow while floating freely in medium), and altered colony formation. Various subpopulations of pancreatic cancer stem cells have been shown to overexpress the genes *CD44*, *CXCR4*, *OCT4*, and *S100P*, which are involved in various aspects of cancer growth and spread. Samples therefore were also analyzed for expression of these genes and their associated proteins. The results showed that, compared with control cells, those cells chronically exposed to cadmium (which the authors dubbed CCE cells) demonstrated characteristics typical of cancer cells.

**Figure:**
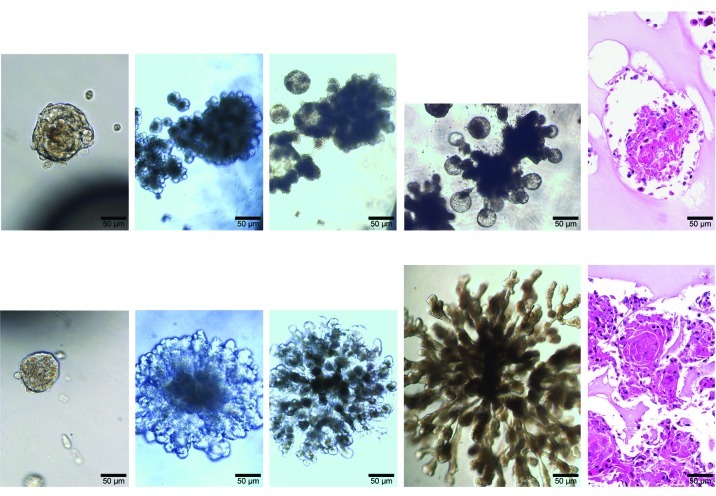
Spheres derived from CCE cells (bottom) rapidly outpaced those derived from control cells (top). Qu et al., doi: 10.1289/ehp.1205082

Stem cells and cancer stem cells, when studied *in vitro*, often form free-floating spheres of cells for reasons not completely understood. The investigators isolated and further cultured spheres generated by control and CCE cells, then submitted them to the same analyses as earlier samples. Spheres derived from CCE cell cultures exhibited cancer cell characteristics and were more numerous and larger than control spheres. They also exhibited increased transcription and expression of *OCT4*, *CD44*, and *CXCR4*. In gel cultures CCE spheres grew aggressively and formed more intricate structures than control spheres; microscopically, these structures appeared malignant, with poorly differentiated cells of many shapes and sizes.

The study shows that *in vitro* exposure of normal human pancreatic cells to a nontoxic level of cadmium chloride can promote the development of cancer characteristics, thus strengthening the evidence for cadmium as a potential cause of pancreatic cancer in humans. Additionally, the study may help investigators define the very early stages of pancreatic cancer, a major advantage for a disease that typically is diagnosed only after it has metastasized.

